# Access to Services in Rural Areas from the Point of View of Older Population—A Case Study in Finland

**DOI:** 10.3390/ijerph16234854

**Published:** 2019-12-02

**Authors:** Ira Verma, Jonna Taegen

**Affiliations:** Department of Architecture, Research Group for Health and Wellbeing Architecture Sotera, Aalto University, 02510 Espoo, Finland; jonna.taegen@aalto.fi

**Keywords:** older people, shrinking municipalities, services, mobility

## Abstract

Independence and having control over one’s own life are important factors for residential satisfaction. In rural areas, the mobility of people is based on owning a private car, due to the lack of public transport. Furthermore, planning in rural municipalities is highly car oriented. Small municipalities with shrinking and aging populations have many challenges to ensure access to services for their residents. This paper focuses on a case study of a small municipality with less than 2000 inhabitants. The objective of the study was to enhance sustainable change in shrinking rural areas and maintain them as good places to live even in the future. Access to local services and social activities is a major challenge for older people, who no longer have the possibility to use their own car. The problem with relocation is the lack of suitable apartments for older people. A dense and walkable municipal centre with accessible apartments may help municipalities provide for their older populations. Moreover, in Finland, second homeowners are an important resource for small municipalities. Spaces for social intercourse between residents and between permanent residents and second homeowners may enhance vitality and community building in these municipalities.

## 1. Introduction

People are concerned about being able to maintain independence and have control over their own life at an old age. These are important factors for life satisfaction [1]. A living environment that supports resident’s functional capacities, enables them to perform their daily activities and access local services forms the basis of an independent life at an old age. In 2015, according to the UN report, 63 per cent of people aged 80 years old and over lived in urban areas [2]. The urban environment with easy access to local services and to public transport can bring many advantages to older people. The spaces for social activities, including cafeterias, shops and cultural events are becoming more important among older people. People of retirement age today represent the first generation used to consuming and travelling [3]. They are wealthier than previous generations and expected to use the power this affords them to choose their living arrangements [4]. Furthermore, as the number of older members of the population in cities is increasing, service providers are starting to adapt their services to this target group [3]. However, many older people do live in rural areas long distances from services and with few transport services. Even though the total population in remote areas is shrinking, by 2040, the percentage of the population 75 years old and over in these areas is projected to increase by 10% [5]. 

Older residents living in rural areas may have a strong attachment to place as they tend to have lived for long periods in the same area. Some of them may want to relocate and move closer to municipal centres, but experience difficulties selling their houses. Falling property values and lack of suitable dwellings in shrinking areas make relocating a challenge. Scott et al. found that older people in rural areas are more likely to move from small, more remote communities to larger communities [6]. These relocations may not be voluntary, however, but may be due to a crisis, or result of an environment that does not support older residents. A Finnish study by Helminen et al. shows that older people are willing to move to the nearest municipal centre if accessible and affordable housing solutions are available [7]. They prefer apartments that are walking distance from local services. However, the shrinking municipalities have little resources to provide new affordable and accessible housing for their older residents. In rural areas, the vacant dwellings in old apartment buildings often lack accessibility and are not suitable for older people. Most of the unoccupied government subsided dwellings (78%) are located in shrinking areas [8] (p.13). Elshof and Bailey argue that shrinkage, and loss of services and population may lead to declining quality of the living environment [8]. Vacant housing and buildings can have a negative effect on the built environment [9]. The planning for shrinkage includes efforts to reduce the number of dwellings. Then again, the municipalities may have difficulties to finance the demolition of these dwellings [10]. 

The social capital and cohesion of a local community are the strengths of small municipalities. According to Rôcak et al., aspects of social sustainability such as interaction with other residents or social networks, participation in collective community activities may support older residents in shrinking areas [11]. Levasseur et al. found that for people in rural areas, length of residency was associated with a sense of belonging to the neighbourhood and with the level of social participation [12]. According to a Finnish survey, the majority of people 75 years old and older have conversations with their neighbours, and social contacts were gaining importance in their residential satisfaction [13] (p. 107). Moreover, many seniors play an active role in the municipality. 

Access to local services and social activities may enhance general wellbeing and a feeling of integration for people in their senior years. According to the FinHealth 2017 study three out of four individuals 80 years old and over self-reported using local services by themselves [14]. However, long distances to services and hindrances in the living environment created challenges especially for this age category. More than half reported to have some physical barriers in their living environment. Earlier findings suggest that the length of residency increased environmental knowledge and enhanced the mobility of older residents [15] (p.181). According to Metz, mobility includes the ability to access people and places that are meaningful, to have physical exercise, and get involved in the local community [16]. Furthermore, the potential to travel, being able to travel, even if the trip is not undertaken, might be important. 

According to a survey, only 4 per cent of people in declining rural centres in Finland use public transport [17] (p.64). Moreover, in rural areas, due to the lack of public transport, the mobility of older people is often dependent on private cars. The oldest age groups, people 75 years old and older, may be forced to give up driving [18] (p. 67), which creates challenges especially for those living in remote areas with long distances to services. Family, friends and neighbours may provide social and practical support to older people in rural areas, and help when public transport is not available [19]. The study by Levasseur et al. indicated that in rural areas, accessibility to key resources, having a driver’s license, children living in the neighbourhood, and more years lived in the current dwelling were factors enhancing older people’s social participation [12]. Shergold and Parkhurst point out, however, that having a car may not resolve all accessibility issues, as these are dependent also on other factors such as health and wealth [19]. According to Kaufmann, mobility is also related to people’s ability, willingness, and their knowledge of how to use transport systems [20] (p. 38). 

Finland has the highest number of second homes in the Nordic countries in relation to population. In the majority of Finnish municipalities, second homes outnumber permanent dwellings [21]. The study by Strandell and Hall observed that recently retired people are the most active users of second homes [13]. Hiltunen and Rehunen found that people of retirement age use second homes more often than younger people do and they stay there longer [22]. The second home culture is currently changing to year-round use and one third of second homes are suitable for winter use. They are used on average 103 days a year. The most popular second home areas are close enough to the larger cities to travel to for a day or weekend [23]. Municipalities generally consider second home users as a positive contributor to the local economy. Second homeowners influence local policy and planning, and may bring economic benefits to municipalities. This can also benefit older people living in remote areas. The challenge is, however, the irregularity and seasonal changes of second home use. Furthermore, second homes are often spread across rural areas and only a few are located in villages [19]. 

## 2. Background

In Finland, population projections show a dramatic drop in birth rates leading to a decreasing population from 2031 [24]. The demographic dependency ratio in rural areas especially is getting higher. Population loss and ageing is affecting the viability of rural communities. The decline of population leads to financial and political transformation where public and commercial services are shifted to larger cities [25]. As a result, the urban structure in small rural centres becomes fragmented, services are missing and buildings are becoming empty [26]. The loss of population and demographic developments have a negative impact on local tax revenues and maintaining services. Strategic long-term concepts are needed to improve the quality of the municipal centres. This paper focuses on an ongoing research and development project in five small municipalities or joint municipalities with less than 10,000 inhabitants. These municipalities in sparsely populated areas with shrinking population have many challenges to manage urban planning and organize their services. The consortium is dealing with questions of urban renewal and sustainable change in sparsely populated areas. This paper presents the results from one of the five municipalities, Pertunmaa. The first case study was implemented in the municipality in 2019. The comparative analyses between all five municipalities will be carried out during the spring 2020. 

The potential to increase the density of the municipal centre with additional housing suitable for older people and a walking friendly environment are the focus of the study. The re-use and shared use of existing buildings are part of the study. In a shrinking community, social capital plays a major role. The densification of municipal centres enables the emergence of places for meeting people. Furthermore, new ways of organizing services as a network together with surrounding communities are evaluated. The results of the strategic concepts are always related to the local conditions and characteristics. The Ministry of Social Affairs and Health has acknowledge that there are considerable regional differences in the quality and availability of services, and basic health care does not function well enough in all municipalities. Currently, the care policy for older people promotes living at home with home care services [27]. According to a Finnish survey, most municipalities participating in the survey considered that the lack of affordable and accessible rental apartments in their area was a challenge for organizing services and home care for older people. The supply of suitable apartments in small municipalities is low or the location of housing is remote [28] (p.20). Currently in Finland, there is an oversupply of 24-hour housing services for older people and 1,500 dwellings remain empty [29]. The housing services may be located in remote places or they do not meet the current standards. The oversupply of housing services may also lead to premature move to a care home [30] (p.7). Mainstream rental apartments in small municipalities may be old, lack accessibility and are not suitable for older people [28] (p.20). Moreover, for example, the renovation of owner-occupied housing for low-income residents is challenging. Enhanced mobility of residents as well as the potential for organizing mobile services are studied together with the residents and various local stakeholders.

Older people profit from a walking-friendly environment, short distances to public transport and meaningful destinations within reasonable proximity of the home. Roosenbloom found that over 80 per cent of daily trips less than 0.5 km by older people were done by walking [31]. A walking-friendly environment may increase opportunities for social and physical activities within the living area. The public transport policy in Finland aims to enable people in remote areas to access services in the nearest municipal centre at least twice a week [32] (p.21). The law does not oblige municipalities to provide the service. Because of the lack of public transport, older people who live outside the municipal centre use their own car, taxis or other shared transportation to access services. In Finland, however, older people seem to avoid asking for help from their relatives and neighbours [33] (p. 61). Public transport is an important alternative for older people with limited mobility. Our earlier study of the urban environment showed that most seniors used public transport. However, people in the oldest age groups, 85 years and older, preferred walking or taking a taxi [15]. Roosenbloom found that older people prefer walking before taking a bus [31]. The review of the literature on walking-friendly environments by Talen and Koschinsky revealed that sometimes housing design that focuses on affordability economizes via walkability [34]. On the other hand, they found evidence that walkability increased property values and the value of place more generally. Initiatives focusing on the improved walkability of municipal centres and innovative transport solutions are needed in rural municipalities to enable people to live there even in the future. The development of age-friendly features may have shared benefits for other groups within the wider population in addition to older people [6] (p. 350). This is important, as rural areas need to have residents of all age groups, to have economic viability and services.

Second homeowners are identified as a potential resource for remote municipalities. An earlier study by Hiltunen and Rehunen found that over half of second homeowners and users in Finland had previously lived in the countryside, and had childhood experiences from rural areas [22]. However, less than one per cent thought of moving permanently to the second home. Czarnecki and Sireni point out that the relationship between permanent residents and second homeowners is important in developing a positive impact on the local economy [35]. They found that municipal actors seem to evaluate the positive impact of second homeowners on infrastructure and services higher than the permanent residents. Kietäväinen et al., point out, however, that the municipal administration may exclude them from local institutions, and treat them as only partial members of the community [36]. The participation of second homeowners in local activities and development projects may bring benefits to the municipality. Many of them have roots in the area and have a strong attachment to place [21]. They may contribute their knowledge and skills to the development of the municipality.

## 3. Aim

The overall objective of the study was to find ways to enhance the livability of the municipal area and improve the wellbeing of residents in rural areas. The goal is to keep these small municipalities good places to live in even in the future. This paper focuses on housing and daily mobility from the point of view of older people in rural areas. The research questions were what are the housing options for older people and how do they access to local services and social activities in rural areas. Furthermore, the aim was to discuss the potential of second home owners as contributors and resource for ageing and shrinking municipalities.

## 4. Materials and Methods 

**Figure 1:**
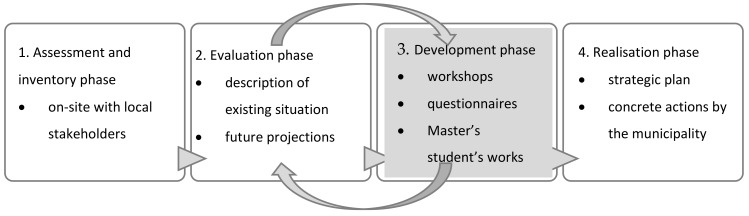
This paper focuses on the results of the third phase of the iterative development process.

The multiple case study method was used to evaluate the living environment in five small municipalities participating in the study. The study sample was chosen according to the size and population structure of the municipalities. The municipalities are located in different parts of Finland. The study used both quantitative and qualitative methods. The statistical data on local population structure, demographic development and population projections formed the basis for the analyses. As part of the study, a questionnaire was prepared and sent to municipal residents to study housing, access to services and mobility in these small municipalities. Moreover, workshops with local residents or other stakeholders were carried out in each of the municipalities. According to Simmons [37] (p. 11) the aim of a case study is to generate an in-depth understanding of a topic and to generate new knowledge for the development of professional practice. In this case the questionnaires and subsequent discussions in the workshops provided an overview of the situation in order to make a strategic plan for improving the municipal urban area and its services (Figure 1).

This paper focus on the results in one of the five municipalities, Pertunmaa. The web survey was open during March and April 2019. A paper format of the questionnaire was sent by post to all residents and people who owned a second home in the municipality of Pertunmaa. This paper focuses on the responses of the senior population, 65 years and older, living or having second homes in the municipality. The residents were asked to report their housing typology, place of residence, and distance to the municipal centre. Moreover, they were asked to evaluate the importance of the local services they use. The questionnaire included questions related to the distance to services and mode of transport. First, the results of the questionnaire and then, a design solution by a Master thesis student in architecture were discussed with the municipal executive board of Pertunmaa. The first meeting was held in May 2019 and the second one in November 2019. The meetings took place at the municipal centre of Pertunmaa.

Defining the contextual background, demographics of the municipality and study sample enable to improve the external validity of this research. The external validity can be achieved by a detailed description of the research [38]. The preliminary results of this study are related to one municipality. The results of such a study can offer only partial view of the problem. However, similar challenges are observed in other small municipalities. Therefore, the results may benefit other municipalities and contribute to their development.

### Study Sample

Both permanent residents and second homeowners in Pertunmaa were invited to participate in the study. The majority of all respondents to the questionnaire (51.6%) were in the age category of 65 years or older (Table 1.). The majority of the respondents in all age categories were second homeowners (60%). The residents (N=229) who responded to the survey represented 13 per cent of the total population of the municipality (25% of the households). Most respondents to the questionnaire that were 74 years and older lived in the centre of the municipality (53%). Single-person households (36%) and two-person households (46%) were the majority. Only 27 per cent in this age category reported that they live alone, but a notable observation was that the majority, 54 per cent of the respondents passed the question about the size of their household. 

## 5. Results

### 5.1. The Municipality of Pertunmaa

The municipality of Pertunmaa is located in the Southern Savonia, approximately 200 km north from Helsinki. The population of the municipality has been decreasing steadily during the last decades. Today, less than 1800 inhabitants live in Pertunmaa, of which 37 per cent are 64 years and older, and 16 per cent, 74 years and older. In the coming years, the population prognostics show a decrease of population that is getting older. By 2030, almost half of the population is expected to have reached retirement age (Table 2). In the summer, however, the population of the municipality doubles with the addition of second homeowners to reach approximately 3700 inhabitants. The second homeowners are regarded as a potential resource for the local economy and the vitality of the municipality. They may, however, have different needs and aspirations in regard to the municipal environment and service provision than the permanent residents. Due to seasonal changes in Finland, second homes are mainly used in the summer. 

**Figure 2:**
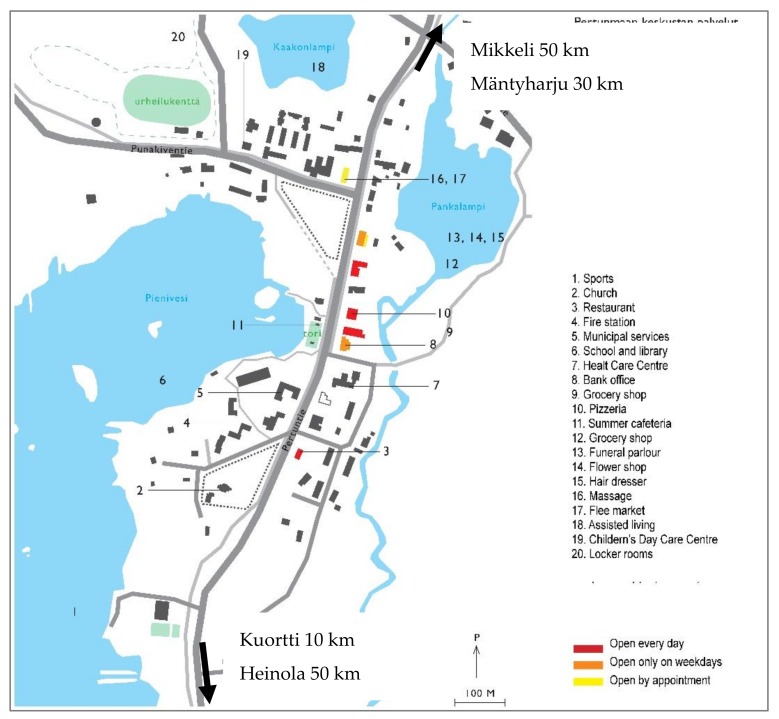
Plan view of the centre of the municipality.

Observations of the urban form show that the planning of small municipalities is highly car oriented. There are also problems such as the lack of pavement and street lighting in the municipal centre. The municipality of Pertunmaa has two distinct commercial service areas. The local services, municipal services and housing for older people are located in the municipal centre. Moreover, the library, school and day care centre for children are in the municipal centre. In the summer, a cafeteria and an open market place (tori) are found by the lake (Figure 2). A shopping area, Kuortti, has been developed 10 km (10 min drive) from the municipal centre near the motorway. There is no public transport in the municipal centre nor between the two centres. The development of two distinct service areas is weakening the urban structure of the municipal centre and creating challenges for the mobility of the older population. This seems to be quite a common development in all five rural municipalities participating in the study.

### 5.2. Housing

Older people tend to reside for longer periods in the same living environment than younger people. The majority of older people living permanently in the area reported that they had lived there for more than 5 years. Statistical data shows that the majority of all residents in Pertunmaa (70%) live in single-family houses (Table 3). Residents 75 years and older live in semi-detached houses and terrace houses (31%) slightly more often than residents on average. 

**Figure 3:**
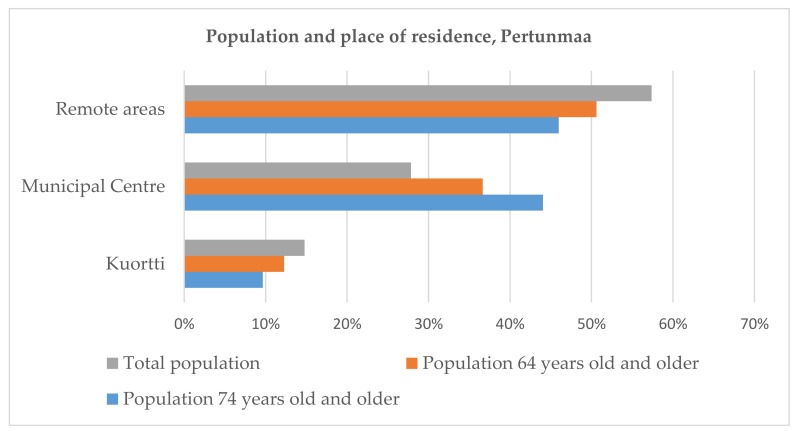
In Pertunmaa, the majority of residents live outside municipal center (OSF, 2017).

Furthermore, statistical analyses reveal that only minority of the population live in Kuortti and the tendency of living near to the municipal centre is increasing with age (Figure 3). The majority of all respondents living permanently in Pertunmaa reported that they live more than 5 km from the centre of the municipality (56%). Only 28 percent of all permanent residents and 38 percent of the residents 64 years old and older responding to the questionnaire reported to live in the municipal centre. 

There are vacant rental row houses in the municipal center that would need major renovations. However, the municipality does not have the resources to renovate them. The apartments does not meet the quality requirements of potential tenants. Recently, the municipality has sold some of the apartment buildings to private developers who have been renovating and renting them out. The results have been successful. 

### 5.3. Use of Services

**Figure 4:**
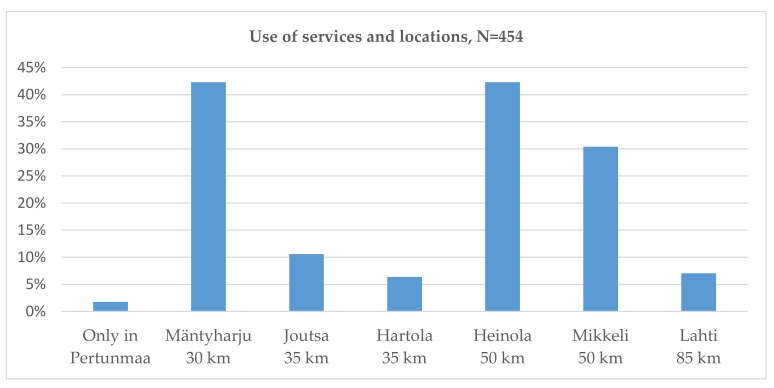
Individuals that reported using the services outside Pertunmaa.

The respondents in all age categories reported that they use local services equally in the municipal centre (89%) and in Kuortti (88%). However, less people 64 years and older reported that they use services in Kuortti (68%). Some essential services, such as the pharmacy and petrol station, however, were located there. The respondents in all age categories used health care services and cultural services in the municipal centre or in larger towns further away. A few persons reported that they use only local services in Pertunmaa. Most respondents reported that they visit larger towns, such as Mikkeli and Heinola, approximate 50 km away, and the smaller municipal centre Mäntyharju 30 km from Pertunmaa (Figure 4). Many reported that they visit these towns weekly or at least monthly. The lack of public transport to these towns was considered a problem. Furthermore, services like the swimming pool and cinema were available only in the larger towns, as was the dentist and optician, for example.

### 5.4. Mobility

**Figure 5:**
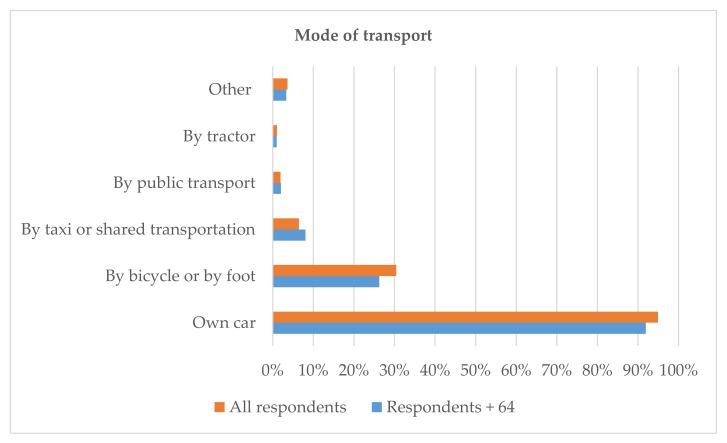
Mode of transport reported by the residents.

The car was the main mode of transport to access services for all respondents (Figure 5). The majority of respondents 74 years and older reported that they have one car (75%) in the family. However, 12 per cent of them reported that they did not own a car. The second most common mode of transport among this age category was bicycle or walking (26%). Ten of the older respondents reported using a taxi or other shared transportation, and only two reported getting a ride with neighbours. Both residents and second homeowners wished to have transport services between the municipal centre and Kuortti, and a few respondents proposed a safe bicycle lane between the two centres. Furthermore, a transport connection to the larger towns of Heinola and Mikkeli was lacking. Several respondents reported that they would use more public transport services if they existed.

### 5.5. Local Development Needs

**Figure 6:**
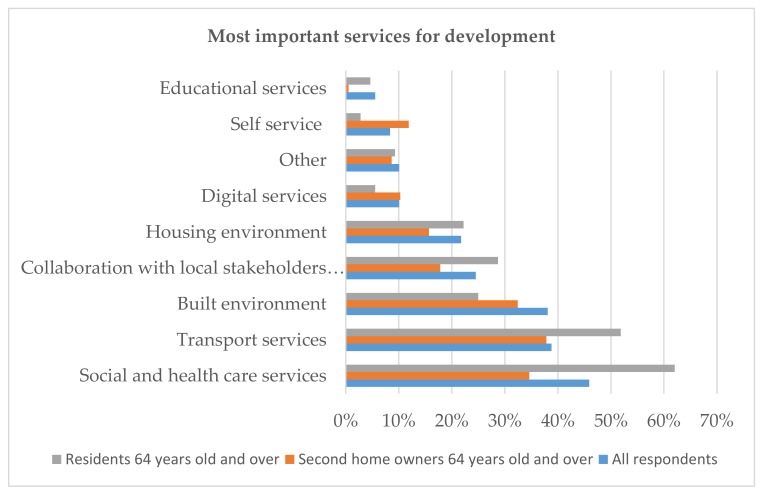
The services that should be developed reported by the residents.

The respondents were asked to choose the three main factors that were most important for the development and vitality of the municipality. The permanent residents and second homeowners highlighted partly the same development factors (Figure 6). The permanent residents 64 years and older found that the social and health care services (62%), public transport services (52%) and collaboration with public services and local associations (29%) were the main development tasks. They were concerned about the social cohesion of the community. The second homeowners 65 years and older reported the social and health care services (38%), public transport services (35%), and the built environment (32%) were the most important areas needing development. They appreciated the natural and idyllic image of the municipality. However, only a minority of the permanent residents considered developing the built environment (25%) and housing environment (22%) important. Furthermore, digitalization has been seen as having potential for remote areas [40]. Our study shows that digital services were only considered important by ten per cent of all respondents. In the age category 65 years and older, 6 per cent of local residents and 10 per cent of the second homeowners considered the development of digital services important.

The municipal executive board pointed out, that most of the services that the second homeowners wished to have in Pertunmaa, already existed there. The services were currently scattered around the municipal centre. Therefore, the navigation and visual guidance between the services should be developed. Moreover, the roads and walking paths would need improvement. Both the older people and the second homeowners pointed out the need for common spaces, living rooms, where to meet other people. The buildings are rarely planned to serve both the needs of inhabitants and second homeowners. 

Residents 75 years and older reported that nature, safe living environment and friendly people were the best features in their area. The second homeowners reported that they appreciated the idyllic landscape and clean natural environment. In the summer, the weekly open market in the centre of the municipality was the space for social activities. It was the most important meeting point between permanent residents and second homeowners. This activity supported the community and involvement of second homeowners. These types of community centres should be enforced to increase possibilities for social interaction between residents. A clear municipal centre and a community centre have been identified as the most important features in the built environment to enhance the vitality of the municipal centre. Furthermore, the respondents were interested in longer hours for services, more cultural events and activities. In a small municipality, they can all be concentrated in one place. This would also benefit older people who are less mobile. 

## 6. Discussion

Older respondents reported that they appreciated the clean natural environment, safety and local community. They valued the strong community and friendly people in their area. However, they were concerned about the diminishing supply of services and the long distance to services. One study by Vuorinen revealed that older people living in a shrinking village associated the slowing down of village life with their own ageing process [41] (p. 172). Scott et al. point out that the economic situation and provision of services is becoming a challenge in rural areas if there is not a cross-section of generations [6]. When municipalities have less resources they need to prioritize their development actions. Therefore, community building, the residential satisfaction of current residents and opportunities for participation may be key tasks for small municipalities. The aim is to keep remote areas attractive to live in also in the future. Therefore, services need to be targeted to a heterogenic population. 

A dense municipal urban structure and a clear community centre with various activities can strengthen social cohesion. Urban development is not the only factor that can resolve the challenges in remote municipalities. Successful actions have been identified in relation to the densification of municipal, commercial and cultural services and the re-use of existing buildings in the municipal centre (e.g., Aschersleben, Germany; Idom-Råsted, Denmark). Moreover, the improvement of walking conditions in the centre of the municipality, in the green areas, and along idyllic waterfronts may enhance the attractiveness of the space and the mobility of residents. As Gehl points out, the space between buildings becomes meaningful only when there are people using it [42]. In urban design, a walking distance of 0.5 km [43] or 5 min [44] is considered a short enough distance that people choose to walk. In Finland, 22 percent of people living in rural areas live within 500 m distance from local services [43]. Furthermore, housing suitable for older people should be located within walking distance of services. Currently in Pertunmaa, approximately 37 percent of residents 75 years old and older live within 500 m walking distance from local services [45]. Hoekstra and al. point out that the need for affordable housing in areas with declining population is expected to be growing. Therefore, the planning for shrinkage should not lead to the lack of affordable rental dwellings for the older people in these areas [10]. In Pertunmaa, for example, there is an empty commercial facility in front of the municipal building that could be refurbished for housing. New housing in the center of the municipality would improve the access to services for older population.

Access to local services is a major challenge for older people who can no longer use their own car. Older respondents regarded that local transport connections to the municipal centre would enhance access to services. Some areas in Europe (e.g., Brieselang, Brandenburg, Germany) have been able to improve access to services and the feeling of inclusion using innovative transport services for older people living outside the municipal centre. Furthermore, the goals of sustainable development aim at reducing the number of private cars. The challenge in rural areas is the lack of other alternatives. Ridesharing or other local initiatives may be successful solutions for rural areas. A recent study in Finland argued that differences in mobility are related to the transport networks and land use rather than the demographic variations between areas [46]. Furthermore, the automation of car traffic is expected to develop in the coming years. It may help some older people in remote areas who do not have private cars. However, the frailest of them would still need help and assistance to leave their home and access a smart vehicle. 

New innovative ways of service delivery in these areas are needed. Digital services have been identified as a potential solution for shrinking remote areas. However, the results of the questionnaire reveal that residents themselves did not consider remote services as an important area of development. Antikainen et al. note that digital services have to be developed from the local perspective to avoid the risk of digital exclusion [39]. Small municipalities can concentrate their services in one central service hub, where the mobile and digital services are delivered to those who otherwise would not have access to them. A local digital hub enabling remote work opportunities may also enhance local economic activity. Moreover, a common living space in the municipal centre may promote social contact between local people and second homeowners. In Pertunmaa, the municipal executive board set out the objective: “We don´t want any new walls in Pertunmaa”. As a result, a design proposal for converting the municipal building to a common living room was presented. The location in the centre of the municipality makes it ideal for social activities for residents, second homeowners and visitors.

Many second homeowners have family roots or other strong ties to the second home location and the cottages are inherited from parents or grandparents. According to Rinne and al. the second homeowners are rarely active in local associations or participating in decision making [46]. They come to enjoy the natural environment and may have a strong interest in preserving the nature. However, many of them are seniors, and need the same municipal services as permanent residents. Moreover, the permanent residents and second homeowners have similar needs for example for transport services and remote technology [47]. In Finland, however, the right to collect local taxes only exists in relation to the municipality of permanent residence. Therefore, demographic development and urbanization causes many challenges to small municipalities. There are some discussions about sharing tax revenues between the municipalities of permanent and second home location. Otherwise, opportunities for Ageing in Place and delivering services in remote municipalities with shrinking populations is becoming difficult. Future developments need to be based on the local situation, and adopt an intergenerational approach that takes account of local resources and strengths. The densification of the urban structure together with new models of governance where the residents take part in the decision-making may support the vitality of these rural municipalities.

## 7. Conclusions

The potential of rural areas is in a strong community. Actions related to planning and service development should contribute to social cohesion within the community. A dense municipal centre with core services – local services, mobile and digital services - may promote vitality and enhance the inclusion of older residents within the community. Moreover, future developments need to adopt an intergenerational approach taking account of all local resources and strengths, including second homeowners. Both the older people and the second homeowners pointed out the lack of spaces for social activities. The idea of combining the social needs for both user groups would need further studies. Moreover, the older people and their housing needs should not be treated separately from the urban development of the municipalities. The development of a walkable municipal centre with accessible and affordable apartments may help older people to remain living in their familiar surroundings as integral part of the community.

## Figures and Tables

**Table 1:** Number of respondents in Pertunmaa (N = 575).

	Total	Male	Female	Residents	Second Homeowners
All respondents	575	272	303	229	349
Respondents 64 years old and older	297	166	131	108	185
Respondents 74 years old and older	113	67	46	45	67

**Table 2:** Total population, population 64 and 74 years old and older for 2018 and projections for 2030^1^.

	Total Population 2018	Total Population 2030	Population 64 Years Old and Older, 2018 N (%)	Population 64 Years Old and Older, 2030 N (%)	Population 74 Years Old and Older, 2018, N (%)	Population 74 Years Old and Older, 2030, N(%)
Pertunmaa	1750	1472	644 (37%)	673 (46%)	280(16%)	391 (27%)

^1^ Official Statistics Finland, OSF, 2019.

**Table 3:** Pertunmaa, household size, age of the oldest resident and housing type ^2^.

	Pertunmaa All	(%)	Residents 74 Years Old and Older	(%)
Single-family house	636	(70 %)	139	(64 %)
Row house, semi-detached house	235	(26 %)	68	(31 %)
Apartments	11	(1 %)	4	(2 %)
Other	28	(3 %)	7	(3 %)

^2^ Official Statistics Finland, OSF, 2018 [39].
